# Cancer vaccines

**DOI:** 10.1054/bjoc.2000.1217

**Published:** 2000-04-27

**Authors:** A G Dalgleish

**Affiliations:** Division of Oncology, Department of Cellular and Molecular Sciences, St George's Hospital Medical School, Cranmer Terrace, Tooting, London, SW17 0RE, UK


*British Journal of Cancer* (2000) **82**(10), 1619–1624
© 2000 Cancer Research Campaign
DOI: 10.1054/ bjoc.2000.1217, available online at http://www.idealibrary.com on IDEAL®

Millennium mini-review

### Cancer vaccines

**AG Dalgleish**

Division of Oncology, Department of Cellular and Molecular Sciences, St George's Hospital Medical School, Cranmer Terrace, Tooting, London SW17 0RE, UK

Vaccination refers to the procedure pioneered by Edward Jenner (EJ), whereby the immune system is primed to respond rapidly to an infectious agent, by exposing it to the important antigens of the infectious agent by using heat-killed or attenuated non-pathogenic versions. EJ used the biologically naturally attenuated (in humans), cow pox to provide resistance to small pox. A cancer vaccine is designed to induce an immune response in a patient who has developed cancer resistant to conventional treatment cancer or who has a high probability of developing a recurrence following adequate treatment. It is reasonable to regard cancer as an infection that has infected the host without inducing an appropriate immune response. The reasons that cancer cells are able to evade the immune system or an effective immune response include:

1. They look like self and bear self-antigens.
2. They actively anergise the immune system to any possible differentiating (between self and tumour) antigens.
3. They may create an immunosuppressive environment which, in addition to secreting a number of immunosuppressive factors, also includes the possession of the equivalent of 'anti missile – missile systems', such as tumour expression of *fas*-L which can induce apoptosis of any incoming tumour antigen specific killer cell (reviewed by Gilboa, 1999; Pawelec, 1999; Walker et al, 1997).

A number of historical observations strongly suggest that an appropriate immune response is associated with tumour regression. These include:

1. William Coley's observation (over 100 years ago) that severe septicaemia/erysipelas was occasionally associated with regression of a number of solid tumours.
2. Spontaneous regression of melanoma deposits associated with infiltrating immune cells (often mentioned – rarely observed!).
3. The ability to occasionally mimic this activity with cytokine administration, such as interleukin-2 and interferon as well as cell-based and peptide-based 'vaccines'.
4. The association between anti-leukaemia activity and the development of graft versus host (GVH) disease in post-bone marrow transplant leukaemia patients. The GVH effect can clearly be separated from the GV leukaemia effect thus allowing for therapeutic extrapolation (reviewed by Browning and Dalgleish, 1996; Dalgleish, 1996; Vile et al 1996).

More recently, the discovery of a board range of antigens which if delivered appropriately can induce anti-tumour responses, coupled with an increased understanding of immunological tolerance, anergy and ignorance, and how to overcome them, has given us another opportunity to try to harness the immune response against cancer and to continue where others left off (Boon et al, 1997; Wang and Rosenberg, 1999).

Coley's observation that potentially fatal bacterial infections could induce an effective anti-cancer response in patients with partially resected tumours led to the development of safer toxins based on the isolated bacteria cell wall preparations which became known as Coley's toxins (Coley, 1991; Nauts and McLaren, 1990). Coley's successes were not readily reproducible by others, and his treatment proved controversial, leading to nearly two decades of detailed development. Essentially, minor changes in manufacture, dosage and scheduling appeared to be the difference between success and failure, which is just as likely to apply to today's attempts to develop cancer vaccines. Coley's toxins were superseded by radiotherapy and subsequently chemotherapy following his retirement. Enthusiasm for immunotherapies and cancer vaccines has waxed and waned nearly every decade or so throughout the 20th century, with initial enthusiasm being dampened by objective clinical studies.

The BCG vaccine has been used to induce anti-cancer immune responses nearly as long as it has been used against TB (Nathanson, 1979). In randomized studies it appears to have a small but not significant difference in the treatment of leukaemia, melanoma and prostate cancer (Cascinelli et al, 1989; Guinan et al, 1982; Nathanson et al, 1979; Tan and Ho, 1993; Vuvan et al, 1978; Zuthrie et al, 1980). Its success as an intra-tumoural agent in the case of melanoma is more impressive, with 20–60% (depending on which study), of tumours disappearing. Unfortunately, no significant effect on non-injected metastases is seen in most cases. Nevertheless, the effect of intravesicular BCG in superficial bladder cancer is dramatic and the treatment of choice over chemotherapy by many urologists. It is thought to act by inducing a specific inflammatory response (Alexandroff et al, 1999; Hurle et al, 1999).

The concept of using tumour cells to treat cancer is far from new and goes back at least to the 17th century! Throughout the last five or more decades, both autologous and allogeneic cell lines have been used to try to treat of number a cancers, particularly melanoma, renal and colorectal (Morton et al, 1992; Vermorken et al, 1999). Autologous tumour preparations appear to be preferred (as they have been in the new era of gene therapy, involving transfection of cytokines, etc, into cells), with allogeneic cell lines being tried when the difficulty of raising autologous lines from enough patients becomes insurmountable. Many studies using cells also use BCG as an adjuvant (Browning and Dalgleish, 1996; Vile et al, 1996). A large number of studies can be briefly summarized, in that early studies often showed anecdotal responses, which failed to translate to significant numbers in larger randomized studies. Responses tend to be associated with

*Received 17 December 1999*
*Accepted 7 January 2000*

intra-dermal administration (as opposed to subcutaneous) and multiple vaccination protocols (as opposed to one or two injections). Of all the approaches along these lines, the one developed by Dr Donald Morton has endured through several decades to the point where it is now in large multi-centred randomized controlled trials. The protocol started with BCG and different allogeneic cells, finally culminating in a three cell-line allogeneic vaccine given initially with BCG and monthly thereafter, in patients with metastatic melanoma. Prior to randomization, experience with the triple-cell vaccine was gained with several hundred patients giving a two- to three-fold increase in survival at 2 and 5 years. This was even more pronounced in those patients who could be rendered disease-free by surgical excision (Hsueh et al, 1998*a*; 1998*b*; Morton et al, 1992). Our own group has recently confirmed this trend in a smaller phase II study which has now been closed, with the introduction of the double-blind randomized placebo control studies for stage III and stage IV patients with metastatic melanoma (Maraveyas et al, 1999*b*).

Two developments have led to a resurgence of interest in developing new cancer vaccines. These are:

1. The ability to transfect cell lines with a variety of immunomodulatory enhancing genes such as cytokines and co-stimulatory factors.
2. The identification of a number of new tumour specific/ associated antigens which can be purified for therapeutic use.

The vast majority of all the early anti-cancer gene therapy studies involve the transfection of mainly interleukin-2 and more recently GM-CSF into autologous cells. In a number of animal models, it has clearly been shown that a variety of genes transfected into the autologous cells will enhance an anti-tumour immune response, and these include most of the cytokines (with a few exceptions), growth factors such as GM-CSF, co-stimulatory factors such as CD 80, CD 86 as well as the expression of HLA class I and class II. Like their earlier counterparts, however, most investigators have been frustrated by the difficulty in obtaining available autologous cells and have looked towards using allogeneic cell lines. Nevertheless, although the majority of investigators try to match the allogeneic cell lines in order to have the same HLA class I as the autologous tumour, our own studies have shown that allogeneic cell lines can confer and enhance an anti-tumour activity compared with autologous cells (Knight et al, 1996; Souberbeille et al, 1996). It is possible that allogeneic cells are also inducing some form of graft-versus-tumour activity. The induction of cross-reactive CTL activity is particularly compelling (Kayaga et al, 1999), a full understanding of which could lead to enhanced exploitation in order to develop more effective allogeneic-based therapeutic strategies.

The number of antigens that have been identified as being more specific to tumour than normal cells has been recently increased dramatically. Prior to this, clinical studies were mainly focussed on gangliosides and mucins. A large number of new antigens have been identified using CTLs from patients who had evidence of immunological responses to identify peptide sequences which may be used as therapeutic targets; a technique pioneered by Boon and colleagues (Boon et al, 1997). Another approach known as 'SEREX' involves the use of serum from responding patients to target humoural epitopes (Tureci et al, 1997).

There are a number of terminologies to define tumour antigens. The most accurate description overall is tumour associated antigens (TAA) which involves a number of onco-fetal developmental antigens which may be expressed: tumour developmental antigens (TDA), e.g. CEA. Some antigens are more specific to tumours than normal cells, such as tumour specific antigens (TSA), which can include exogenous viruses that drive the tumour e.g. EBV and lymphoma, HPV and cancer of the cervix, as well as mutations of endogenous genes such as oncogenes, e.g. *ras* and suppressor genes, e.g. P53 (see Table 1).

**Table 1** Tumour antigens

| |
|---|
| **Tumour-specific antigens (high affinity, no tolerance)** |
| 1. Viral antigens associated with tumour pathogenesis |
| EBV – lymphoma, nasopharyngeal cancer |
| HPV – cervix, anal (several other sites suspected) |
| HBV/HCC – hepatoma (liver cancer) |
| 2. Mutation antigens |
| Specific tumour and not other tissues |
| *ras*, p53 bcr/abl, CDK-4, Caspase 8 |
| **Tumour-associated antigens (wide spectrum of affinity and tolerance)** |
| 1. Cancer testes (CT) antigens, restricted to: |
| Primitive germs cells of the testes and following activation of expression in a number of tumours |
| MAGE-1–3, GAGE, BAGE, RAGE, PAGE, NY-ESO-1 and many similar others |
| 2. Differentiation antigens: normal tissues with altered expression on tumour cells |
| MART-1/Melay A, gp100, tyrosinase, TRP-1/2 GM2 ganglioside |
| HER-2/neu, CEA, MUC: |

NB This list is exemplary only, several hundred antigens have now been described

A full description of the tumour antigens described to date is beyond the scope of this review. However, it is worth noting that gangliosides and mucins which are qualitatively and quantitatively differentially expressed on cancer cells represent good 'mono' antigen targets. They are the subjects of large randomized studies in melanoma and breast cancer respectively, following encouraging phase II trials. (Livingstone et al, 1994; MacLean et al, 1996). Many other tumour antigens can be reduced to peptides and presented with adjuvants or given with autologous dendritic cells (DCs) grown ex-vivo. Whatever antigen is identified or selected, it is unlikely to induce much of an immune response given alone.

It has been known for many years that in order to induce an immune response it is necessary to use an adjuvant which covers a broad range of candidates from Freund's adjuvant, the TB Vaccine, Bacillus Calmette-Guerin (BCG) through to alum, the latter of which is the only one with a full licence in humans. Polly Matzinger would argue that adjuvant is the necessary 'danger' signal necessary to alert the immune system which does not perceive most antigens delivered subcutaneously as dangerous, and hence will not 'react' to them (Fuchs and Matzinger 1996). With most tumour antigens being essentially derived from self, the method of presentation and the type of immune response induced is crucial. The immune response can be predominantly cell-mediated with IL-2, γ-IFN and IL-12 being preferentially induced known as a Th-1 response, or predominantly humoural-mediated with IL-4, IL-5, IL-6 and IL-10 induction, a Th-2 response. It has been suspected for some years that cell-mediated responses are deficient in many cancer patients and more recently that humoural-dominant responses may be detrimental to some cancers in this regard. There is now increasing evidence that this is the case in advanced cancer patients (Goto et al, 1999) including melanoma and prostate cancer (Maraveyas et al, 1999a; Hrouda

 *© 2000 Cancer Research Campaign*

**Table 2** Clinical trials – melanoma

| Randomized | Stage ( ) Vaccine | Control | Status |
|---|---|---|---|
| Livingston | (III) GM2-BCG | BCG | No significant benefit for GM2 |
| ECOG | (III) GM2-Q21 | HD/IFNα | In progress |
| Progenics | (IIA) GM2-QS.21 | Observe | About to start |
| | (III) GM2-QS.21 | Placebo | Cancelled |
| Morton PMCV | (III) Three allogeneic cell line + | BCG + Placebo | In progress |
| | (IV) BCG | BCG + Placebo | In progress |
| Ribi Melacine | (IV) Two cell lines + Detox (adjuvant) | Chemotherapy | Equivalent |
| Hersey | (IIB) (III) Allogeneic lysate 1 cell | Untreated | In progress |
| Byrstryn | (III) 4 allo (1 xeno) cells lysates | Untreated | Small numbers; significant bias in favour of vaccine |

et al, 1998). In addition, we have documented a marked inhibition of the Th-1 responses in patients with early colorectal cancer (Duke's stages A and B) which reverts following surgical removal (Heriot et al, *in press*). Alum, historically the most commonly used adjuvant, is a Th-2 preferential inducer and hence is probably not ideal for use in most cancer vaccines. Adjuvants which give a predominate Th-1 response include BCG (which can paradoxically induce a Th-2 in some people), other mycobacterium such as *M. Vaccae* (known as SRL-172), oil/water emulsions (e.g. IDEC-AF) as well as quil-saponin mixtures (e.g. QS-21). Most of these adjuvants work best by intradermal as opposed to subcutaneous injections, as they are taken up by special dendritic cells which then migrate to the lymph nodes, where they activate the relevant T cells.

In addition to inducing T-cell responses it would appear that the isotype of the humoural response is also important. In the melanoma whole-cell vaccine of Morton and colleagues there is a clear survival advantage with a dominant IgM (to gangliosides and the TA90 antigen) response, which is lost if the IgG becomes dominant (Hsueh et al, 1998*b*) (this is totally the opposite of what would be expected if vaccinating against an infectious agent). There are a large number of different adjuvants in pre-clinical studies and these include cytokines such as IL-2 and IL-12 which may be given exogenously as well as being transfected into tumour cells.

In addition to adjuvants, enhanced immune responses can be made using ex vivo cultured (DCs) derived from GM-CSF and IL-4 and/or TNFα supplemented cultures following pulsing with antigens prior to re-infusing into patients' veins or lymph nodes (Bjorck, 1999). Once again, the details of this approach are critical as it is also possible to actively tolerize against antigens using dendritic cells DCs (Ludewig et al, 1999). Antigens present on cells (i.e. autologous and allogeneic cell-based vaccines) can be rendered more immunogenic by transfecting with stimulatory molecules, e.g. B7-1, cytokines such as GM-CSF, IL-2, etc, as well as combinations thereof. Viral vectors (such as adenovirus) can be used to present known antigens (e.g. CEA) with cytokine inserts, e.g. IL-2, either incorporated in a viral vector or as a DNA vaccine. Both these approaches can then use cultured DCs as the presentation vehicle, with cells being presented as lysates, and DNA (or RNA) being transfected directly into the DCs. DCs are the optimal APC and attempts have been made to form hybridomas to create an APC full of tumour antigens. Although possible with B cells and some tumour cell lines, stable fusions are more difficult with DCs (Scott-Taylor et al, *in press*). However, further research may enable this approach to be reduced to practice.

Another interesting approach under the gene therapy label is the transfer of the HSVtk gene into the tumour using an adenoviral vector, for instance. Following administration of ganciclovir which is converted by HSVtk-driven phosphorylation of ganciclovir into toxic by-products, an immune-mediated bystander response is induced in addition to local cell-to-cell transfer of the toxic by-products. The enhanced immune response is of particular interest in developing vaccine-based protocols. A number of similar gene prodrug combinations are also being pursued in this area in addition to HSVtk/ganciclovir (Perry et al, *in press*).

Clinical trials have thrown up a number of problems in inducing an effective anti-tumour immune response. These include:

1. The lack of induction of an appropriate cell-mediated response (which may be limited by the patient's own immunogenetics, especially in the case of peptides).
2. The need for recruitment of effective helper pathways to expand the initial response, e.g. IL-2-dependent pathways.
3. The lack of development of an appropriate effector response, e.g. CTL and/or NK response.
4. The presence of a strong immunosuppressor environment (e.g. the secretion of IL-10, TFG-β, and the expression of *fas*-L) that down-regulates any attacking immune response.

Even if an appropriate antigen-specific response is induced, it may not last long due to the absence or poor induction of a memory response. It has been noted with frustration that effective vaccines need to be given regularly, e.g. monthly, in the presence of even minimal residual disease. However, even in the absence of tumour-produced immunosuppressive factors, this is likely to be due to the fact that most tumour antigens are 'self' (or very close), and that although it is possible to break tolerance, the ability to readily induce a long-term memory T-cell response would be to induce potentially catastrophic autoimmunity. It is of note that current 'cancer vaccines' rarely induce significant auto-immunity even when given with strong adjuvants, other than vitiligo patients with melanoma (a phenomenon which occurs in 5–10% of the population who are not vaccinated). It is therefore necessary to consider regular vaccination in a therapeutic setting, although caution is necessary as it is possible to over-stimulate and 'burn out' the immune response with some agents such as BCG, which can destroy any beneficial immune response if given too frequently.

How much antigen with which adjuvant, at which site, how often, for how long, with what other therapy, for which tumour (or sub-type) are all unknown quantities in determining an ideal

*© 2000 Cancer Research Campaign*

**Table 3** Examples of phase II clinical studies in melanoma

| Peptides | Transfected cells | |
|---|---|---|
| MAGE | Auto/allo + | IL-2 |
| MART | | GM-CSF |
| GP100 | | B7 |
| Tyrosinase | | IL-4 |
| Peptides + IL2 | | Auto + Allo HLA-B7 |
| Peptides + dendritic cells | | Combinations |
| Antigens in virus vectors | | eg. IL-2 + B7 |
| Antigens as DNA | | |
| Non-specific; SRL-172 | | |

**Table 4** Other clinical trials/studies

| | Phase | Status |
|---|---|---|
| **Other vaccine-based studies:** | | |
| Prostate: Cells (auto/allo) + GM-CSF | | IP |
| Peptides + DC | | IP |
| Allo cells + adjuvant | | IP |
| Gangliosides | | IP |
| **Colorectal studies/pancreas** | | |
| Autologous cells + BCG | | SR [Ref] Vermorken et al, 1999 |
| Viral vector + CEA | | P |
| 105 AD7 | II & III | NS |
| Cells and adjuvant | | P |
| *Ras* mutant peptides + DCs | | IP |
| **Breast cancer:** | | |
| Sialyl Tn/MUC-1. IP. (peptides and in vectors) | | IP |
| Neu/HER and anti-idiotype | | IP |
| **Lung** | | |
| Anti-GD3 (BEC + BCG) | III EORTC | IP |
| Chemo + SRL-172 | III | IP |
| Autologous cells | II | IP |
| HLA matched allogeneic cells | | |
| **Renal** | | |
| SRL-172 | II | IP |
| Autologous cells | | IP |
| DCs + cell lysates | | IP |

**Legend:** IP = in progress; P = planning stage; SR = significant response; NS = non-significant response.

vaccine strategy. It is clear that minor changes in the starting conditions can lead to big changes in the outcome, a fact that has led to speculation elsewhere about applying the ‘chaos theory’ to vaccine development (Dalgleish 1999). Moreover, if these objectives were readily achieved it may then be necessary to attack the anti-immune defences of the tumour which include targets such as TGF-β and IL-10 production and excess CD55 (DAF) and *fas*-L expression etc. These approaches may result in additive if not synergistic effects in outcome.

Optimization of such approaches requires the use of a reliable laboratory model. The majority of cancer vaccines to date have been based on the demonstration of anti-tumour efficacy in animal models. A large number exist and only the naturally non-immunogenic models pose a major challenge for protection and Therapeutic strategies, an example is the B16 melanoma model whose F10 cell line represents a difficult challenge to block immunologically. Most successful experiments on these models are protective, in that vaccination has to take place before a tumour challenge is undertaken. Therapy studies whereby vaccination commences after tumour challenge are a much harder hurdle to overcome. We have used several of these models to demonstrate that allogeneic cell vaccination is able to induce stronger protective responses than autologous cell vaccines (Knight et al, 1996; Hrouda et al, *submitted*; Souberbielle et al, 1996; 1998). This effect extends to the two known rat prostate cancer models, Dunning and the Lobund-Wistar, whereby cells from the former confer an 80% protection in the latter (Hrouda et al, *submitted*). These models have also been used to demonstrate that optimal adjuvants can perform as well as cytokine transfected cells (Souberbielle et al, 1996) and that GM-CSF transfected allogeneic cells can induce an effective therapeutic response by inducing cross-reacting CTL response (Kayaga et al, 1999). Other encouraging approaches include heat-shock protein/tumour antigen complexes (Srivastava and Udonon, 1994), tumour-cell antigen-presenting cell hybridomas, DNA and RNA vaccines given intramuscularly or transfected into dendritic cells, as well as antigens given as peptides or encoded in a viral/bacterial vector which may also be given with DCs.

A large number of clinical trials involving cancer vaccines are currently ongoing (see Tables 2, 3 and 4) Many of these have focused on melanoma, where encouraging phase II studies have led to randomized trials for gangliosides and whole allogeneic (Morton MCV) cell lines. Trials with single peptides have been disappointing and multiple peptides are more encouraging, although ‘help’ in the form of IL-2 or presentation with DCs appears to be required in most cases (Rosenberg et al, 1998). As so many melanoma (and other cell-type tumour antigens) are shared with other tumour types, widespread application of these approaches to other tumours, such as renal, prostate, lung, breast, pancreas and colorectal, are being tried. However, the general principles established in pre-clinical and early melanoma studies will probably continue to apply, in that the immune response is difficult to induce in advanced disease and cancer vaccines work best in the adjuvant or minimal residual disease setting. The two most intensive areas of expansion regarding clinical trials at the present time are the use of dendritic cells and the role of DNA/RNA vaccines. They can, of course, easily be combined with RNA/DNA transfection of DCs already in progress. The ex vivo growth of DCs (with at least three possible progenitor types) inherently carries the potential for considerable variability following culture in GM-CSF plus IL-4/INFα. Many questions remain unanswered, such as the optimal time to harvest, what to pulse with and which route to use for presentation, e.g. intravenous or intranodal. Occasional dramatic responses have been seen (in a number of tumour types) which are not reliably reproducible, and the reasons for this require further elucidation. The role of the CD40 and FLT-3 ligands look like an increasingly important area of research in this regard (Di Nicola et al, 1998; French et al, 1999; Siena et al, 1995). DNA vaccines allow for the bespoke manufacture of private idiotypes as seen in lymphomas. A recent study has shown that such vaccinations given to bulk reduced, yet ‘lymphoma PCR-positive patients’ could be rendered PCR-negative in 8/11 lymphoma patients (Bendandi et al, 1999). Previous studies in advanced disease were disappointing due to the marked immunosuppression seen in patients with advanced lymphomas.

It will, therefore, be necessary to integrate cancer vaccines into a sequentially managed therapy (SMT) programme to treat cancer patients, which will include the use of other modalities such as radical surgery and chemotherapy for debulking disease and even

 *© 2000 Cancer Research Campaign*

the incorporation of radiotherapy to enhance autologous tumour necrosis, and hence antigen presentation to stimulate the immune system. Vaccines may help other treatments to work better than alone. For instance, our own experience regarding the enhanced susceptibility of vaccine patients with melanoma to radiotherapy, which was thought to be an original observation until several such reports were uncovered (Cameron et al, 1990). Hence, our means of assessment will need to change regarding the use of vaccines used to treat patients with non-debulkable disease, where the lack of complete and partial responses often gives way to static disease, improved quality of life and prolonged survival, which within such programmes are realistic goals for non-toxic outpatient treatments. Cytokines and monoclonal antibodies may beneficially be incorporated into SMT, which in the case of Herceptin could be complemented by ongoing anti-idiotype vaccination against the HER receptor.

Most solid cancers arise out of areas of chronic inflammation, which are histologically if not clinically evident, e.g. lung, oesophagus, stomach, liver, colorectal, cervix, etc. Chronic inflammation whether or not induced by cigarettes, helicobacter, hepatitis B, bile salts, etc, is associated with a depression of Th-1 responses and an increase in angiogenesis, both features of wound healing and pregnancy. Such a state allows immunological sanctuary for the obvious reasons that if cell-mediated responses were not suppressed in these conditions, T-cells would react with self tissues with resultant autoimmunity, and in the case of pregnancy the fetus would be rejected. Long-term Th-1 suppression allows oncogen mutations to survive as they are not immediately eliminated by CTLs, and the segmental progression to a malignant cell can progress unchecked (O'Byrne et al, *in press*). The enhanced angiogenesis can enable distance spread and prolong immunosuppression. Thus if this is indeed the case in vivo, cancer vaccines may well benefit from co-treatment with anti-angiogenic treatments and anti-inflammatory agents. There are obvious implications for prevention. One fact that supports this speculation is the unexpected drop in the incidence of colorectal cancer in patients taking daily aspirin (Thun, 1997).

### REFERENCES

Alexandroff AB, Jackson AM, O'Donnell MA and James K (1999) BCG immunotherapy of bladder cancer: 20 years on. *Lancet* **353(9165)**: 1689–1694

Bendandi M, Gocke CD, Kobrin CB, Benko FA, Sternas LA, Pennington R, Watson TM, Reynolds CW, Gause BL, Duffey PL, Jaffe ES, Creekmore SP, Longo DL and Kwak LW (1999) Complete molecular remissions induced by patient-specific vaccination plus granulocyte-monocyte colony-stimulating factor against lymphoma. *Nat Med* **5(10)**: 1171–1177

Bjorck P (1999) Development of dendritic cells and their use in tumour therapy. Clin Immunol **92(2)**: 119–127

Boon T, Coulie PG and Van den Eynde B (1997) Tumour antigens recognised by T cells. *Immunol Today* **18(6)**: 267–268

Browning M and Dalgleish AG (1996) Introduction and historical perspective. In: *Tumour Immunology – Immunotherapy and Cancer Vaccines (Cancer, Clinical Science in Practice)*. Dalgleish AG, Browning M (eds) Cambridge University Press: London and New York

Cameron RB, Spiess PJ and Rosenbery SA (1990) Synergistic antitumour activity of tumour-infiltrating lymphocytes, interleukin 2, and local tumour irradation. Studies on the mechanism of action. *J Exp Med* **17(1)**: 249–263

Cascinelli N, Rumke P, Mackie R, Morabito A and Bufalino R (1989) The significance of conversion of skin reactivity to efficacy of bacillus Calmette-Guerin (BCG) vaccinations given immediately after radical surgery in stage II melanoma patients. *Cancer Immunol Immunother* **28(4)**: 282–286

Coley WB (1991) The treatment of malignant tumours by repeated inoculations of erysipelas. With a report of ten original cases, 1893. *Clinical Orthopaedics* **262**: 3–11

Dalgleish AG (1996) The case for therapeutic vaccines. *Melanoma Research* **6**: 5–10

Dalgleish AG (1999) The relevance of non-linear mathematics (chaos theory) to the treatment of cancer, the role of the immune response and the potential for vaccines. *QJM* 347–359

Di Nicola M, Siena S, Bregni M, Longoni P, Magni M, Milanesi M, Matteucci P, Mortarini R, Anichini A, Parmiani G, Drexler I, Erfle V, Sutter G and Giannni AM (1998) Gene transfer into human dendritic antigen-presenting cells by vaccinia virus and adenovirus vectors. *Cancer Gene Ther* **5(6)**: 350–356

French RR, Chan HT, Tutt AL and Glennie MJ (1999) CD40 antibody evokes a cytotoxic T-cell response that eradicates lymphoma and bypasses T-cell help. *Nat Med* **5(5)**: 548–553

Fuchs EJ and Matzinger P (1996) Is cancer dangerous to the immune system? *Semin Immunol* **8(5)**: 271–280

Gilboa E (1999) How tumours escape immune destruction and what we can do about it. *Cancer Immunol Immunother* **48(7)**: 382–385

Goto S, Sato M, Kaneko R, Ioth M, Sato S and Takeuchi S (1999) Analysis of Th1 and Th2 cytokine production by peripheral blood mononuclear cells as a parameter of immunological dysfunction in advanced cancer patients. *Cancer Immunol Immunother* **48(8)**: 435–442

Guinan P, Toronchi E, Shaw M, Crispin R and Sharifi R (1982) Bacillus calmette-guerin (BCG) adjuvant therapy in stage D prostate cancer. *Urology* **20(4)**: 401–403

Heriot AG, Marriott JB, Cookson S, Kumar D and Dalgleish AG (2000) Reduction in cytokine production in colorectal cancer patients: association with stage and reversal by resection. *Br J Cancer: (in press)*

Hrouda D, Todryk SM, Perry MJA, Souberbielle BE, Kayaga J, Kirby RS and Dalgleish AG (2000) Allogeneic whole rumour cell vaccination in the rat model of prostate cancer. *Br J Urol: (submitted)*

Hrouda D, Baban B, Dunsmuir WD, Kirby RS and Dalgleish AG (1998) Immunotherapy of advanced prostate cancer: a Phase I/II trial using *Mycobacterium vaccae* (SRL-172). *Br J Urol* **82**: 568–573

Hsueh EC, Famatiga E, Gupta RK, Qi K and Morton DL (1998*a*) Enhancement of complement-dependent cytotoxicity by polyvalent melanoma cell vaccine (Cancer Vax): correlation with survival. *Ann Surg Oncol* **5(7)**: 595–602

Hsueh EC, Gupta RK, Qi K and Morton DL (1998*b*) Correlation of specific immune responses with survival in melanoma patients with distant metastases receiving polyvalent melanoma cell vaccine. *J Clin Oncol* **16(9)**: 2913–2920

Hurle R, Losa A, Manzetti A and Lembo A (1999) Intravesical bacille Calmette-Guerin in Stage T1 grade 3 bladder cancer therapy: a 7-year follow-up. *Urology* **54(2)**: 258–263

Kayaga J, Souberbielle BE, Shiekh N, Morrow WJW, Scott-Taylor TH, Vile R and Dalgleish AG (1999) Anti-tumour activity against B16-F10 melanoma with a GM-CSF secreting allogeneic tumour cell vaccine. *Gene Therapy* **6**: 1475–1481

Knight BC, Souberbielle BE, Rizzardi GP, Ball SE and Dalgleish AG (1996) Allogeneic murine melanoma cell vaccine – a model for the development of human allogeneic cancer vaccine. *Melanoma Research* **6(4)**: 299–306

Livingston PO, Wong GY, Adluri S, Tao Y, Padavan M, Parente R, Hanlon C, Calves MJ, Helling F and Ritter G (1994) Improved survival in stage III melanoma patients with GM2 antibodies: a randomized trial of adjuvant vaccination with GM2 ganglioside. *J Clin Oncol* **12(5)**: 1036–1044

Ludewig B, Odermatt, Ochsenbein AF, Zinkernagel RM and Hengartner H (1999) Role of dendritic cells in the induction and maintenance of autoimmune diseases. *Immunol Rev* **169**: 45–54

MacLean GD, Reddish MA, Koganty RR and Longenecker BM (1996) Antibodies against mucin-associated sialyl-Tn epitopes correlate with survival of metastatic adenocarcinoma patients undergoing active specific immunotherapy with synthetic STn vaccine. *J Immunother Emphasis Tumor Immunol* **19(1)**: 59–68

Maraveyas A, Baban B, Kennard D, Rook GAW, Westby M, Grange JM, Lydyard P, Stanford JJ, Jones E, Selby P and Dalgleish AG (1999*a*) Possible improved survival of patients with Stage IV AJCC melanoma receiving SRL 172 immunotherapy: Correlation with induction of increased levels of intracellular interleukin 2 in peripheral blood lymphocytes. *Ann Oncol* **10.7**: 817–824

Maraveyas A, Compton L, Dunleavey R, Sage D, Naverette C, Mortion D and Dalgleish A (1999*b*) 3-Fold increase in survival for stage IV melanoma patients treated with MCV allogeneic vaccine: Confirmation of previous phase II data. *Eur J Cancer* **35(4)**: 356

Morton DL, Foshag LJ, Hoon DS, Nizze JA, Famatiga E, Wanek La, Chang C,

Davtyan DG, Gupta RK and Elashoff (1992) Prolongation of survival in metastatic melanoma after active specific immunotherapy with a new polyvalent melanoma vaccine. *Ann Surg* **216(4)**: 463–482
Nathanson L (1979) Immunotherapy of melanoma. *J Cutan Pathol* **6(3)**: 213–226
Nathanson L, Schoenfeld D, Regelson W, Colsky J and Mittelman A (1979) Prospective comparison of intralesional and multipuncture BCG in recurrent intradermal melanoma. *Cancer* **43(5)**: 1630–1635
Nauts HC and McLaren JR. Coley Toxins – the first century. *Adv Exp Med Biol* **267**: 483–500
O'Byrne KJ, Dalgleish AG, Browning MJ, Steward WP and Harris AL (2000) Angiogenesis and suppression of cell mediated immunity precede and play a central role in the pathogenesis of malignant disease. *Eur J Cancer (in press)*.
Pawelec G (1999) Tumour escape from the immune response: the last hurdle for successful immunotherapy of cancer? *Cancer Immunol Immunother* **48(7)**: 343–345
Perry MJA, Hrouda D and Dalgleish AG (2000) Prospects for vaccination in prostate cancer. *Drugs Aging: (in press)*
Rosenberg SA, Yang JC, Schwartzentruber DJ, Hwu P, Marcincola FM, Topalian SL, Restifo NP, Dudley ME, Schwarz SL, Spiess PJ, Wunderlich JR, Parkhurst MR, Kawakami Y, Seipp CA, Einhorn JH and White DE (1998) Immunological and therapeutic evaluation of a synthetic peptide vaccine for the treatment of patients with metastatic melanoma. *Nat Med* **4(3)**: 321–327
Scott-Taylor TH, Pettingale R, Clarke I, Stuhler G, Lararth MC, Walden PM and Dalgleish AG (2000) Human tumour and dendritic cell for hybrids generated by electrofusion: potential for cancer vaccines. *Biochem Biophys Acta: (in press)*
Siena S, Di Nicola M, Bregni M, Mortarini R, Anichini A, Lombardi L, Ravagnani F, Parmiani G and Gianni AM (1995) Massive *ex vivo* generation of functional dendritic cells from mobilized CD34+ blood progenitors for anticancer therapy. *Exp Hematol* **23(14)**: 1463–1471
Souberbielle BE, Knight BC, Morrow WJW, Darling D, Fraziano M, Marriott JB, Cookson S, Farzaneh F and Dalgleish AG (1996) Comparison of IL-2 and IL-4 transfected B16-F10 cells with a novel oil-microemulsion adjuvant for B16-F10 whole cell tumour vaccine. *Gene Therapy* **3(10)**: 853–858
Souberbielle BE, Westby M, Ganz S, Kayaga J, Mendes R, Morrow WJW and Dalgleish AG (1998) Comparison of four strategies in the B16-F10 melanoma model. *Gene Therapy* **5(11)**: 1447–1455
Srivastava PK and Udonon H (1994) Heat shock protein-peptide complexes in cancer immunotherapy. *Curr Opin Immunol* **6(5)**: 728–732
Tan JK and Ho VC (1993) Pooled analysis of the efficacy of bacille Calmette-Guerin (BCG) immunotherapy in malignant melanoma. *Journal of Dermatological Surgery and Oncology* **19(11)**: 985–990
Thun MJ (1997) Aspirin and gastrointestinal cancer. *Adv Exp Med Biol* **400A**: 395–402
Tureci O, Sahin U and Pfreundschuh M (1997) Serological analysis of human tumour antigens: molecular definition and implications. *Mollecular Medicine Today* **3(8)**: 342–349
Vermorken JB, Claessen Am, Van Tinteren H, Gall HE, Ezinga R, Meijer S, Scheper RJ, Meijer CJ, Bloemena E, Ransom JH, Hanna MG Jr and Pinedo HM (1999) Active specific immunotherapy for stage II and stage III human colon cancer: a randomised trial. *Lancet* **353(9150)**: 345–350
Vile R, Souberbielle B and Dalgleish AG (1996) Tumour Vaccines. In: *Immunotherapy in Cancer*, Gore M and Riches P (Eds), John Wiley & Sons Ltd: Chichester
Vuvan H, Fiere D, Doillon M, Martin C, Coiffier B, Felman P, Bryon PA, Favre-Gilly J and Revol L (1978) BCG therapy in acute non lymphoid leukaemias. *Scandinavian Journal of Haematology* **21(1)**: 40–46
Walker PR, Saas P and Dietrich PY (1997) Role of Fas ligand (CD95L) in immune escape: the tumour cell strikes back. *J Immunol* **158(10)**: 4521–4524
Wang RF and Rosenberg SA (1999) Human tumour antigens for cancer vaccine development. *Immunol Rev* **170**: 85–100
Zuhrie SR, Harris R, Freeman CB, Maclver JE, Geary CG, Delamore IW and Tooth JA (1980) Immunotherapy alone vs no maintenance treatment in acute myelogenous leukaemia. *Br J Cancer* **41(3)**: 372–377

 *© 2000 Cancer Research Campaign*

